# Twisting and constriction of a HeartMate II driveline in the area of a repair site

**DOI:** 10.1007/s10047-020-01181-0

**Published:** 2020-05-30

**Authors:** Sven Maier, Friedhelm Beyersdorf, Christoph Benk

**Affiliations:** Department of Cardiovascular Surgery, Heart Center Freiburg University, Faculty of Medicine, University of Freiburg, Hugstetter Strasse 55, 79106 Freiburg, Germany

**Keywords:** Left ventricular assist device, Driveline repair, Driveline exchange

## Abstract

The driveline’s durability is crucial for optimal long-term support with a left-ventricular assist device (LVAD). The incidence of percutaneous driveline fracture after HeartMate II LVAD implantation is low. For the first time, we describe a patient with an already repaired driveline and a massive constriction and twisting of the driveline in the area of the repair site. This dramatic finding necessitates a renewed exchange of the external part of the driveline by the manufacturer. Due to the increasing number of patients with elongated LVAD support, the stability of the driveline and possible repairs including the replacement of the driveline are becoming more and more important. Our case report describes a possible serious late complication after replacement of the driveline, shows possible risks for this development, and describes the necessity of a prophylactic X-ray examination of repaired drivelines to detect such complications as early as possible.

## Introduction

The driveline’s durability is crucial for optimal long-term support with a left-ventricular assist device (LVAD). The incidence of percutaneous driveline fracture after HeartMate II LVAD implantation is low [1, 2]. Exchanging the external part of the driveline by the manufacturer or repairing it with special tape is the option during long-term LVAD therapy if there are lead fractures or damages in the driveline’s covering. During LVAD support, most lead fractures occurred in the external part of the driveline [3, 4].

## Case report

Our 77-year-old male patient underwent implantation of a HeartMate II LVAD 3998 days before due to dilative cardiomyopathy. Implantation of the driveline was done using the double-tunnel driveline technique and the driveline leaves the skin near the midline in the direction of the left lateral abdominal wall [5]. There occurred neither revisions nor transfer of the driveline exit site. Furthermore, the patient had no history of driveline infection or debridement around the driveline exit site. 1197 days after implantation, the whole external part of the driveline was replaced approximately 35 cm from the exit site by the manufacturer due to a cable break. The length of the repaired driveline corresponds to the length of the original driveline.

After this replacement, several repairs were performed due to clefts in the driveline with special tape “ResQ-tape” (Tape Innovation GmbH, Rheinbach, Germany). The patient used an ungrounded cable for 1056 days as a precaution due to the previous driveline repairs. Current status of the patient is NYHA class 2–3, body mass index of 27.8 kg/m^2^, and body surface area of 2.19 m^2^. It is possible for him to go for a walk and do light gardening.

**Fig. 1:**
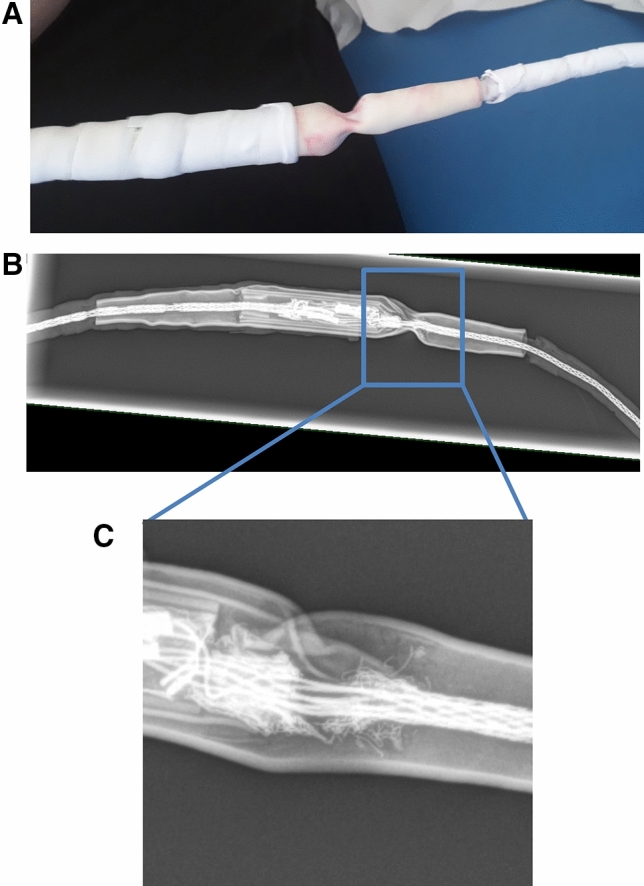
**a** Repaired driveline after removal of ResQ-tape with constriction, **b** X-ray of the constriction, **c** extension with cleft in the shield

Now, he presented with a kink in the driveline in the area of the repair site in our outpatient clinic. After removing the tape in this area of the driveline, we detected a twist and extremely reduced driveline diameter. We splinted that part of the driveline to stabilize it, and took an X-ray of the driveline, which revealed a major constriction in the driveline in the area of the repaired site, as well as a cleft in the shield (Fig. 1). No alarms occurred at the LVAD controller. The data analysis by the manufacturer showed no sign of a defect of the leads in the driveline. This dramatic finding carrying the acute risk of a lead fracture meant that the external driveline had to be exchanged again. One day later, the manufacturer replaced the whole external driveline including the repair site, leaving as much of the intact driveline as possible to the driveline exit site.

## Discussion

Exchanging the external part of the driveline by the manufacturer or repairing it with special tape is the option during long-term LVAD therapy if there are lead fractures or damage in the driveline’s covering. Long-term results after exchanging the external part of the driveline were reported in a case series; no complications in long-term follow-up have appeared [1].

Nevertheless, visual and haptic inspection of the repaired driveline, independent of the type of repair, is essential to identify any kinks, twists, or constrictions as soon thereafter as possible. We describe for the first time a twist and massive constriction of a repaired driveline directly at the repair site that necessitated a second driveline exchange.

We suspect two possible mechanisms for our finding: (1) permanent torque of the repaired driveline from wrapping several layers of tape around the driveline during repeated repairs; (2) permanent stress at the same position on the driveline due to the patient’s habitual movements, e.g. storing the bag, rubbing the driveline on clothes, or kinking during motion.

To prevent and possibly detect such twisting and constriction of the driveline, the following measures could be helpful: (1) no winding of the ResQ-tape more than twice to prevent a permanent torque—during additional repairs, the tape should first be removed and then rewound; (2) if possible, avoid winding the ResQ-tape always in the same direction so as to prevent constant torque on the driveline; (3) take a precautionary X-ray of the repaired driveline, e.g. once a year.

**Fig. 2:**
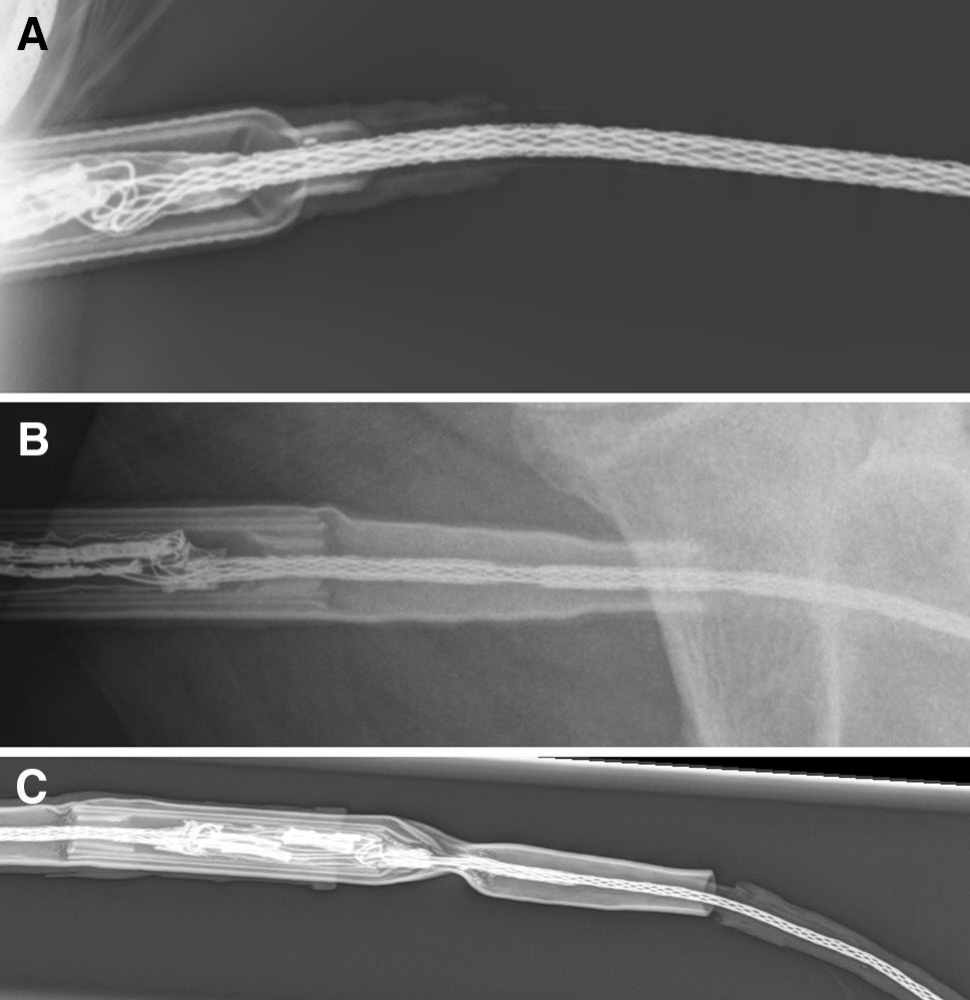
X-ray of the exchanged driveline after **a** 606 days, **b** 1977 days, and **c** 2801 days

If we had X-rayed the repaired driveline of our patient regularly and not just twice in 2800 days and had not carried out in-depth repairs with tape, we would probably have noticed the break in the shield, the kinking and massive constriction of the driveline much earlier (Fig. 2).

Due to the increasing number of patients with elongated LVAD support, the stability of the driveline and possible repairs including the replacement of the driveline are becoming more and more important. Our case report describes a possible serious late complication after replacement of the driveline, shows possible risks for this development, and describes the necessity of a prophylactic X-ray examination of repaired drivelines to detect such complications as early as possible.
