# Correction to: Twisting and constriction of a HeartMate II driveline in the area of a repair site

**DOI:** 10.1007/s10047-021-01280-6

**Published:** 2021-06-14

**Authors:** Sven Maier, Friedhelm Beyersdorf, Christoph Benk

**Affiliations:** Department of Cardiovascular Surgery, Heart Center Freiburg University, Faculty of Medicine, University of Freiburg, Hugstetter Strasse 55, 79106 Freiburg, Germany

## Correction to: Journal of Artificial Organs (2020) 23:371–373 10.1007/s10047-020-01181-0

The article “Twisting and constriction of a HeartMate II driveline in the area of a repair site”, written by Sven Maier, Friedhelm Beyersdorf and Christoph Benk, was originally published Online First without Open Access. After publication in volume 23, issue 4, page 371–373 the author decided to opt for Open Choice and to make the article an Open Access publication. Therefore, the copyright of the article has been changed to © The Author(s) 2020 and the article is forthwith distributed under the terms of the Creative Commons Attribution 4.0 International License, which permits use, sharing, adaptation, distribution and reproduction in any medium or format, as long as you give appropriate credit to the original author(s) and the source, provide a link to the Creative Commons licence, and indicate if changes were made. The images or other third party material in this article are included in the article’s Creative Commons licence, unless indicated otherwise in a credit line to the material. If material is not included in the article’s Creative Commons licence and your intended use is not permitted by statutory regulation or exceeds the permitted use, you will need to obtain permission directly from the copyright holder. To view a copy of this licence, visit http://creativecommons.org/licenses/by/4.0. Open access funding enabled and organized by Projekt DEAL.

The original article has been corrected.

